# Comparing qSMI and qCEUS for assessing vascularization in uterine cervical cancer: operable versus non-operable group

**DOI:** 10.3389/fonc.2024.1380725

**Published:** 2024-08-12

**Authors:** Yi Zhu, Yanjie Li, Yixin Tang, Jie Zhang, Shijun Jia, Zhuolin Jiang, Xinyi Luo, Mitsuya Ishikawa, Tomoyasu Kato

**Affiliations:** ^1^ Department of Ultrasound, Sichuan Cancer Research Center for Cancer, Sichuan Cancer Hospital and Institute, Sichuan Cancer Center, Affiliated Cancer Hospital of University of Electronic Science and Technology of China, Chengdu, China; ^2^ Graduate School, Chengdu Medical College, Chengdu, China; ^3^ Department of Ultrasound, Suining Central Hospital, Suining, China; ^4^ Department of Gynecological Oncology, Sichuan Cancer Research Center for Cancer, Sichuan Cancer Hospital and Institute, Sichuan Cancer Center, Affiliated Cancer Hospital of University of Electronic Science and Technology of China, Chengdu, China; ^5^ Department of Pathology, Sichuan Cancer Research Center for Cancer, Sichuan Cancer Hospital and Institute, Sichuan Cancer Center, Affiliated Cancer Hospital of University of Electronic Science and Technology of China, Chengdu, China; ^6^ Department of Gynecology, National Cancer Center Hospital, Tokyo, Japan

**Keywords:** cervical cancer, superb microvascular imaging, contrast-enhanced ultrasonography, vascular index, time-intensity curves

## Abstract

**Objective:**

The present study aimed to compare the effectiveness and reliability of quantified superb microvascular imaging (qSMI) and quantified contrast-enhanced ultrasonography (qCEUS) in assessing vascularization in both operable and non-operable uterine cervical cancer.

**Methods:**

A case-control study included 64 patients with pathology-proven and untreated cervical cancer, who underwent transvaginal ultrasonography combined with qSMI and qCEUS between January 2022 and June 2023. SMI results were quantified as the vascular index (VI), which were compared to 12 quantitative parameters of CEUS calculated with time-intensity curves (TIC).

**Results:**

According to FIGO staging and different treatment regimens, 64 patients with cervical cancer were divided into operable group (IA ~ IIA, n = 19) and non-operable group (IIB ~ IV, n = 45). In comparison to the operable group, the non-operable group showed significantly higher values in VI, peak intensity (PI), area under the curve (AUC), wash-in area (iAUC), wash-out area (oAUC), wash-in rate (WiR), mean intensity (Mean Int), and standard deviation (STD) (all P < 0.05). VI demonstrated strong correlations with CEUS parameters, notably PI (r = 0.854, P < 0.001) and AUC (r = 0.635, P < 0.001). Furthermore, VI showed a better predictive performance for treatment-group assignment than qCEUS parameters, with an 80.7% accuracy, 64.4% sensitivity and 89.5% specificity.

**Conclusion:**

Both qSMI and qCEUS exhibit significant and comparable utility in detecting microvascular hyperplasia and predicting treatment-group assignments in cervical cancer. Furthermore, qSMI may offer added convenience in implementation.

## Introduction

Cervical cancer is one of the most common malignant tumors in the female reproductive system and remains a leading cause of death among women worldwide. Recent trends indicate an alarming rise in cervical cancer incidence among younger women (1, 2). Cervical cancer develops progressively from precancerous conditions to invasive cancer, and over 99% of cases are attributed to human papillomavirus (HPV) infections (3, 4). While clinical staging currently guides cervical cancer treatments, this approach is not based on surgical or pathological staging, thus introducing potential subjectivity and challenges in precise diagnosis. A crucial concern in the clinical field revolves around enhancing the accuracy of evaluating the size and extent of cervical cancer lesions. This precision is vital for accurate staging, which, in turn, plays a pivotal role in shaping treatment strategies, prognostic assessment, and measuring the effectiveness of therapeutic interventions.

HPV infection is the principal etiological contributor to cervical cancer and CIN, yet the emergence of new blood vessels, known as neovascularization, is crucial in shaping the development and progression of cervical lesions (5–7). We understand that the source and richness of a tumor’s blood supply can, to a certain extent, reflect the tumor’s origin, infiltration scope, and growth rate. In another light, the vascular network of a tumor can be seen as emblematic of the tumor itself. Several studies have shown that tumor angiogenesis is an independent prognostic factor for cervical cancer recurrence and poor prognosis, including disease-free survival and overall survival. Elevated angiogenesis in cervical cancer is associated with a shorter lifespan (8–11). The “gold standard” for characterizing tumor angiogenesis is the immunohistological analysis of microvessel density (MVD) within the tumor. However, this strategy involves invasive procedures, and due to heterogeneity between tumors and within individual tumors, the results can be inconsistent and unreliable (12). Thus, counting intra-tumoral MVD may not be the ideal tool for all clinical objectives, especially when monitoring a tumor’s response to non-surgical treatments.

Color Doppler flow imaging (CDFI) and power Doppler imaging (PDI) are widely used as adjunctive assessments in grayscale ultrasonography. Conventional Doppler imaging provides information about tumor blood flow, aiding doctors in better understanding the biological characteristics of cervical cancer and supporting treatment decisions (13). Additionally, conventional Doppler imaging can also be utilized to monitor changes in intra-tumoral blood flow after treatment, serving as one of the indicators of treatment efficacy (14). However, due to the limited capability of conventional Doppler imaging in displaying low-velocity, low-flow microvessels within lesions, there is a noticeable overlap in Doppler characteristics between benign and malignant tumors.

With advancements in instrument performance and the advent of novel sonographic contrast agents, ultrasonography imaging has effectively enhanced two-dimensional images and Doppler signals of solid organs. This progress allows for the reflection and observation of blood flow perfusion in both normal and pathological tissues. Currently, nearly all ultrasonography diagnostic devices come equipped with built-in time-intensity curve analysis software. This allows for qualitative analysis, including assessments of lesion enhancement patterns, such as the degree and sequence of enhancement, margins, internal uniformity, penetrating vessels, and perfusion defects. Studies have shown that contrast-enhanced ultrasonography (CEUS) and magnetic resonance imaging (MRI) have comparable detection rates for cervical lesions (15), and both can be utilized to evaluate the angiogenic activity of an entire cervical tumor (16, 17). Some scholars believe that functional and dynamic imaging methods might be more appropriate for assessing angiogenic activity in terms of patient survival rates compared to the current histomorphological markers of tumor angiogenesis, such as MVD (18). However, all these imaging methods have their limitations, including the necessity for venous access and potential risks of contrast agent allergies.

Superb Microvascular Imaging (SMI) represents the latest technology in ultrasonography Doppler techniques, emerging from recent advancements in the field. SMI is a sensitive Doppler technique that employs an intelligent filtering system to separate low-flow signals from artifacts. This technology enhances the diagnostic capabilities of grayscale ultrasonography, offering analysis not only for microvascular morphology but also for tumor microvascular perfusion information. Moreover, with the aid of specialized software (the VI Test App from Toshiba Medical Systems Corporation), quantitative analysis of tumor vessels has recently become feasible. The vascular index (%) represents the ratio of Doppler signal pixels to the total lesion pixels. Our preliminary studies suggest that the diagnostic value of qSMI parameters in cervical cancer is notably higher than that in high-grade CIN, with a significant consistency observed among different observers (19). Simultaneously, SMI parameters demonstrate potential in monitoring cervical cancer treatment responses (20). qSMI holds promise as an imaging technique for detecting and characterizing cervical lesions. We anticipate that this parameter will offer valuable insights into the extent of vascularization, complementing the qualitative assessments previously discussed.

**Figure 1:**
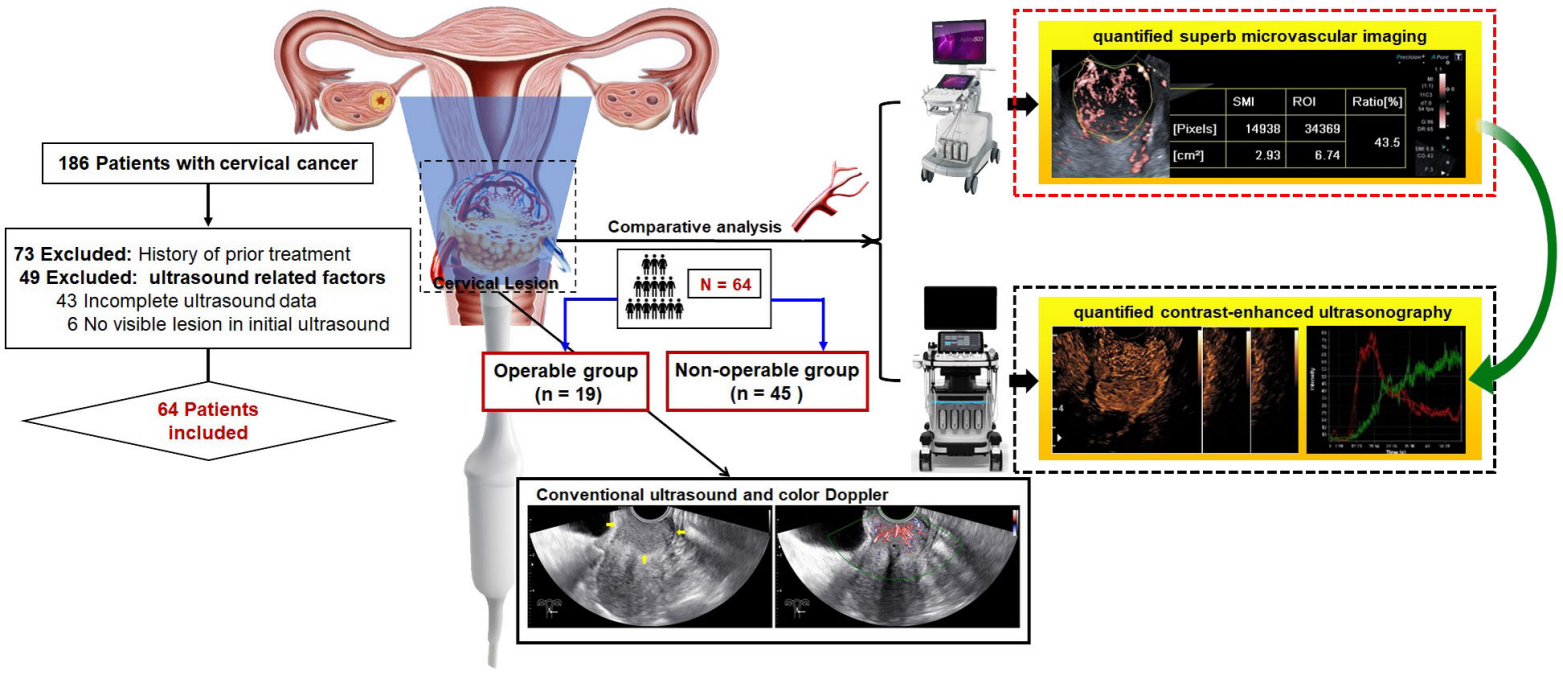
Graph abstract and flow chart of patient enrollment. This study aimed to comparatively analyze the performance of quantified superb microvascular imaging (qSMI) and quantified contrast-enhanced ultrasonography (qCEUS) for assessment of microvasculature in cervical lesions and for prediction of treatment assignment.

In this study, we employed both SMI and CEUS to investigate the perfusion patterns of cervical cancer. We compared the quantitative evaluations of cervical lesion microvasculature and predictive performance for the treatment-group assignment using qSMI and qCEUS (**Figure 1**). As a non-invasive method to assess angiogenic status in cervical cancer, quantitative SMI offers valuable insights for patients with cervical cancer.

## Materials and methods

### Patients

The institutional ethics committee of our hospital approved this retrospective study (Approval No. SCCHEC-02-2022-007). The requirement for informed consent was waived. This case-control study included 64 patients, with a mean age of 52.7 ± 11.3 years (range: 23 - 78 years), between April 2021 and October 2022. All participants underwent real-time transvaginal ultrasonography (TVUS), CEUS, and SMI assessments, followed by histopathological diagnosis through multiple punch biopsy or surgery. The staging of cervical cancer patients was performed according to the FIGO criteria (21). Based on FIGO staging and varying treatment regimens, the patients were further divided into two groups. The operable group consisted of patients with FIGO stages IA to IIA, who underwent surgical treatment. Additionally, those with preoperative tumors larger than 4 cm received platinum-based neoadjuvant chemotherapy (NACT). The non-operable group included patients at advanced stages (FIGO stages IIB to IV), who underwent chemoradiotherapy. Exclusions from the study criteria included patients who had previously undergone treatment, those with incomplete medical records, or those with insufficient ultrasonography imaging (**Figure 1**).

### Ultrasonographic examination

All ultrasonography examinations for the 64 patients were conducted by a single experienced sonographer with 11 years of experience in ultrasonography and 8 years in CEUS. We utilized a Samsung RS80A ultrasonography machine (Samsung Medison Co. Ltd., Seoul, Korea) equipped with a multifrequency 5 ~ 9 MHz endovaginal transducer (V5-9) for baseline ultrasonography, color Doppler flow imaging (CDFI), and CEUS to optimize cervical visualization. The 2D-TVUS scanner employed compound- and speckle-reduction imaging techniques, but did not employ tissue harmonic imaging, offering a maximum probing depth of 18 cm and a sector width angle of 150°. Our primary focus lay in describing the size, shape, and vascular pattern of cervical lesions, which were quantitatively examined using CEUS and SMI.

### Quantified contrast-enhanced ultrasonography

CEUS was conducted following baseline ultrasonography by the same experienced sonographer. SonoVue^®^ (Bracco, Milan, Italy) served as the contrast agent, suspended in 5 ml of saline solution. For each patient, 1.8 ml of SonoVue (Bracco) was administered via the antecubital vein in a bolus fashion (within 1 ~ 2 s), followed by a 5 ml flush of 0.9% physiological saline. The bolus injection was skillfully administered by an operator with 8 years of experience. CEUS was carried out in dual-window mode with a low mechanical index (MI) (0.06 ~ 0.08). The total gain was set at 55 ~ 65 (CEUS), with a dynamic range between 40 ~ 55 dB, depth ranging from 6 ~ 8 cm, and a penetrative frequency. The video recording of the CEUS examination commenced with the bolus injection and was continuously recorded as a DICOM file for 120 seconds, with a focus on the cervical lesions. A region of interest (ROI) was identified for analysis, positioned within the margins of the cervical lesion, avoiding necrotic regions, and defined based on the enhanced area observed during the arterial phase. Twelve time-intensity curve (TIC) parameters, including peak intensity (PI), rise time (RT), mean transit time (MTT), area under the curve (AUC), wash-in area under curve (iAUC), wash-out area under curve (oAUC), time to peak (TTP), fall time (FT), wash-in rate (WiR), wash-out rate (WoR), mean intensity (Mean Int), and standard deviation (STD), were obtained using the TIC quantitative analysis software integrated into the ultrasonography machine. All ultrasonography image analyses were performed by the same sonographer with over 5 years of experience.

### Quantified superb microvascular imaging

SMI was conducted using the Aplio i800 US system (Canon Medical Systems, Tokyo, Japan) equipped with a multifrequency linear 3 ~ 11 MHz endovaginal transducer (Pvt-781vte). Consistent settings were employed for all SMI examinations throughout the study, including an 8.5 cm depth, 3.5 focal zone, 5.8 MHz Doppler frequency, 43 color gain, and a frame rate exceeding 50 fps. These settings were maintained to ensure quantitative US comparisons (19, 20). The Vascular Index (VI) value was derived by manually outlining the lesion boundary in a static SMI image displaying the maximum Doppler signals. VI measurements for the lesions were obtained by a single experienced sonographer with 3 years of experience in SMI.

### Statistical analysis

Statistical analysis was conducted using SPSS software version 26.0 (SPSS Inc., Chicago, IL, USA). Continuous variables were assessed using the Mann-Whitney test, while categorical variables were analyzed using Fisher’s exact test. The correlation between qSMI results with VI and qCEUS parameters with time-intensity curves was determined using Spearman correlation. The correlation coefficient values were interpreted as follows: <0.20 indicated virtually no correlation, 0.21 ~ 0.40 signified weak correlation, 0.41 ~ 0.60 indicated moderate correlation, 0.61 ~ 0.80 represented high correlation, and >0.81 demonstrated greater or very high correlation (22). To assess the predictive value of qSMI and qCEUS for treatment-group assignment in cervical cancer, receiver operating characteristic (ROC) curves and area under the curve (AUC) were employed. A two-sided test was utilized for all analyses, and statistical significance was defined as P < 0.05.

## Results

### Patient population

The 64 cervical cancer patients were stratified into two groups: the operable group (IA ~ IIA, n = 19) and the non-operable group (IIB ~ IV, n = 45). The mean age of patients in the operable group was slightly lower than that in the non-operable group (P = 0.814). According to the FIGO staging system, the distribution was as follows: 1 case was categorized as stage IA, 11 as stage IB, 7 as stage IIA, 6 as stage IIB, 33 as stage III, and 6 as stage IV. Histologically, the cervical cancer cases were classified as 55 cases of squamous cell carcinoma (SCC), 7 cases of adenocarcinoma (AC), 1 case of adenosquamous carcinoma (ASC), and 1 case of neuroendocrine small cell carcinoma (NSCC). **Table 1** presents an overview of tumor characteristics. The SCC-Ag levels and tumor sizes were significantly lower in the operable group than in the non-operable group (P = 0.001 and P < 0.001, respectively). There were no significant differences in age, BMI, smoking habits, personal/family history of tumors, menopausal status, CA125 levels, and histologic types between the two groups (P > 0.05).

**Table 1:** The clinic and demographic characteristics of the patients with cervical cancer in different groups.

	Operable (n=19)	Non-operable (n = 45)	P value
Age (y, Mean ± SD)	49.4 ± 10.4	54.1 ± 11.5	0.116
BMI (kg/m^2^, Mean ± SD)	23.1 ± 2.3	24.3 ± 2.1	0.057
Smoking habit (%)			0.658
Yes	2 (10.5)	5 (11.1)	
No	17 (89.5)	40 (88.9)	
Personal/family history of tumors (%)			0.341
Yes	0	3 (6.7)	
No	19 (100)	42 (93.3)	
Menopause status (%)			0.178
Yes	11 (57.9)	33 (73.3)	
No	8 (42.1)	12 (26.7)	
CA125 [U/ml, M (P_25_ ~ P_75_)]	13.1 (10.0 ~ 19.7)	20.3 (11.9 ~ 34.1)	0.050
SCC-Ag [ng/ml, M (P_25_ ~ P_75_)]	1.0 (0.6 ~ 1.9)	3.7 (1.4 ~ 13.2)	0.001
Tumor size (V)* [cm^3^, M (P_25_ ~ P_75_)]	7.0 (4.0 ~ 15.0)	35.0 (22.0 ~ 63.5)	< 0.001
Histologic type (%)			0.212
SCC	14 (73.7)	41 (91.1)	
AC	4 (21.1)	3 (6.7)	
ASC	1 (5.3)	0	
NSCC	0	1 (2.2)	

*V = d_1_×d_2_×d_3_×π/6.

BMI, Body mass index; SCC-Ag, squamous cell carcinoma-related antigen; SCC, squamous cell carcinoma, AC, adenocarcinoma; ASC, adenosquamous carcinoma; NSCC, neuroendocrine small cell carcinoma.

### Quantified contrast-enhanced ultrasonography analysis (time-intensity curve)

**Figure 2:**
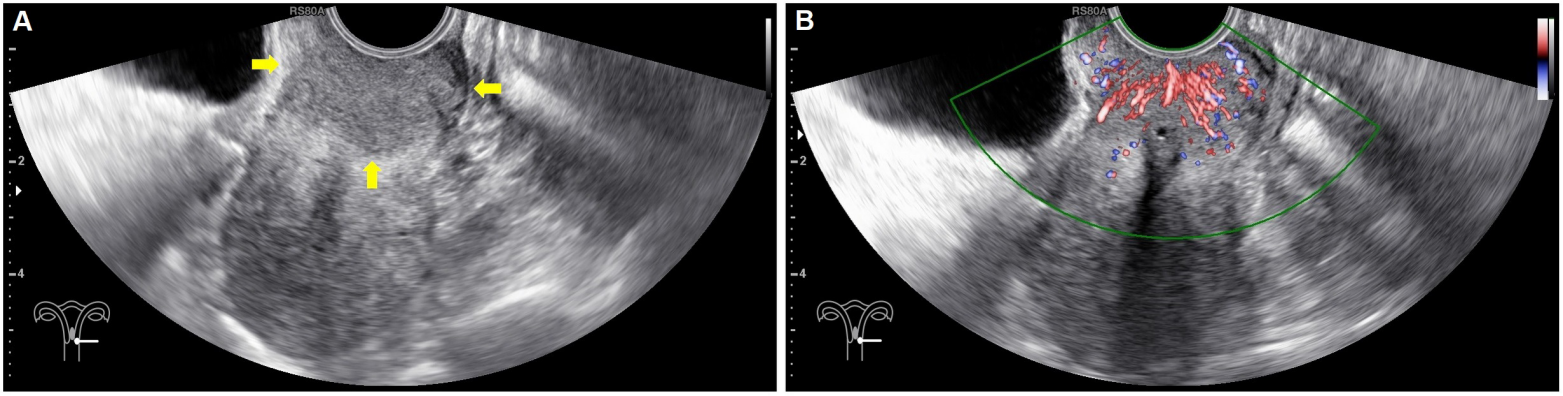
Baseline ultrasonography and color Doppler flow imaging (CDFI) in a 41-year-old with confirmed stage IIB cervical cancer. **(A)** Sagittal gray-scale images of the uterus reveal a hypoechoic, well-defined a lesion (arrows) in the cervix, with tortuosity of the cervical canal and unclear visualization. **(B)** Colour Doppler flow imaging showed spotted intratumoral blood flow signals.

**Figure 3:**
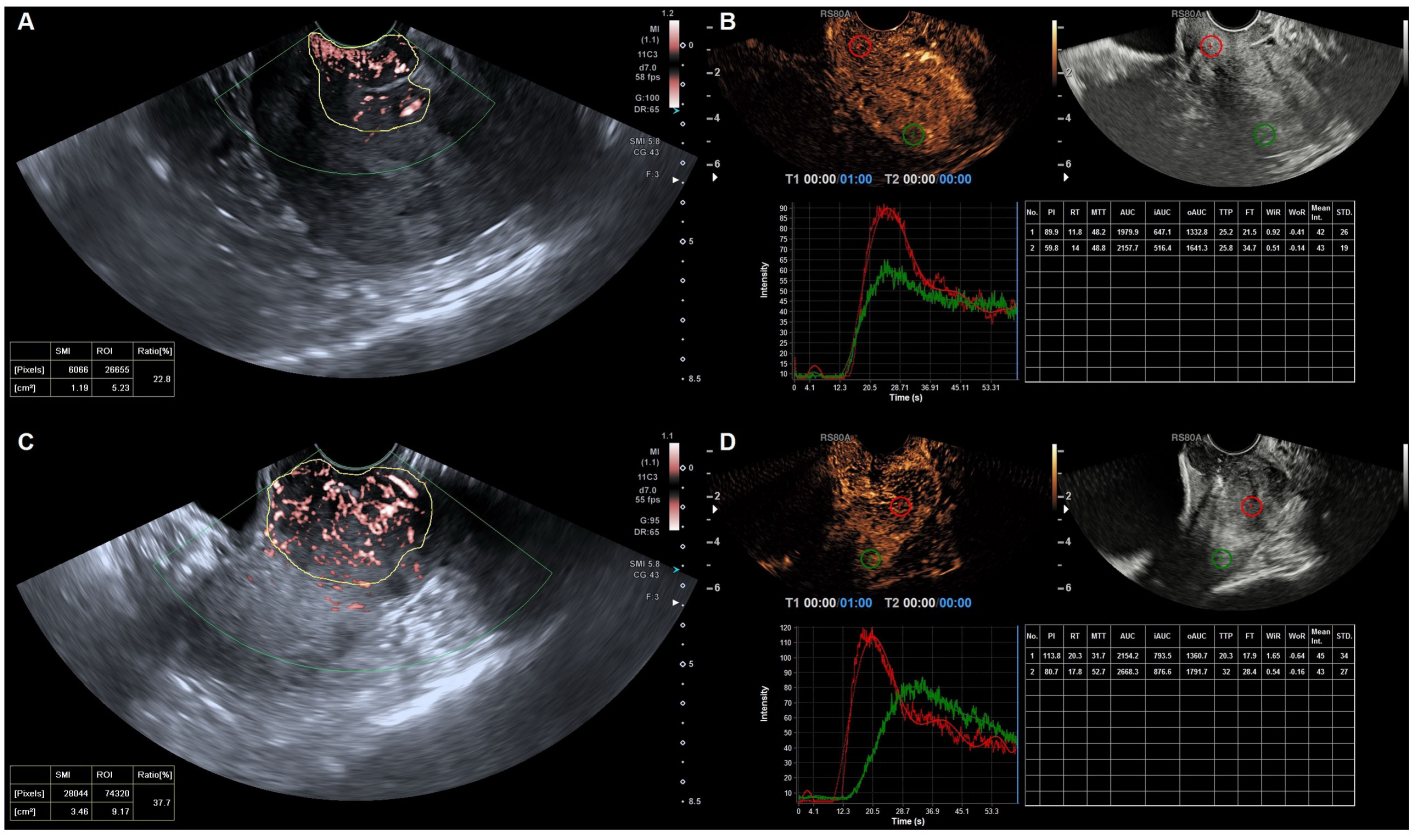
Quantified superb microvascular imaging (qSMI) and quantified contrast-enhanced ultrasonography (qCEUS) in cervical cancer of the different groups. **(A)** qSMI and **(B)** qCEUS in a 52-year-old with confirmed stage IIA1 cervical cancer followed by surgical treatment. **(C)** qSMI and **(D)** qCEUS) in a 56-year-old with confirmed stage IIIC1 cervical cancer followed by chemoradiotherapy. VI was obtained by manually delineating the boundary of the cervical lesion (in yellow) on the SMI images. Output TIC for cervical cancer (in red) and myometrium (in green).

In each case, conventional ultrasonography revealed a mass with varying echogenicity, exhibiting clear and well-defined margins, and an irregular shape, occasionally associated with disruption of the cervical canal. Color Doppler flow imaging (CDFI) demonstrated abundant spot-like blood flow signals within the tumor, particularly in advanced cervical cancer (**Figure 2**). Contrast-enhanced ultrasonography (CEUS) was employed to assess the perfusion of cervical lesions. **Table 2** presents the quantitative variables in the analysis, including reference regions of interest (ROIs). The lesions exhibited higher values in terms of peak intensity (PI), area under the curve (AUC), wash-in area under the curve (iAUC), wash-out area under the curve (oAUC), wash-in rate (WiR), wash-out rate (WoR), mean intensity (Mean Int), and standard deviation (STD), along with shorter values for rise time (RT), mean transit time (MTT), and time to peak (TTP), when compared to the reference regions (myometrium) (all P < 0.05). Furthermore, when compared to the myometrium, 73.7% (14/19) of cervical lesions in the operable group exhibited as well-defined masses with synchronous enhancement during the arterial phase, transitioning into rapidly or synchronously regressing hypoenhanced masses during the venous phase. In the non-operable group, all cervical lesions presented as rapidly hyperenhanced, followed by rapid regression to hypoenhanced masses with well-defined borders (**Figure 3**). As shown in **Table 3**, compared to the operable group, PI, AUC, iAUC, oAUC, WiR, Mean Int, and STD were significantly increased in the non-operable group (all P < 0.05).

**Table 2:** Contrast-enhanced ultrasonography variables in the region of interest (ROI) of the cervical lesions and reference tissues (myometrium).

	Cervical cancer	Reference region	P value
Peak intensity (PI)	97.40 ± 31.43	45.91 ± 20.44	< 0.001
Rise time (RT)	15.79 ± 8.05	21.31 ± 11.40	0.002
Mean transit time (MTT)	42.28 ± 10.04	48.17 ± 11.22	0.002
Area under the curve (AUC)	2492.37 ± 985.67	1528.17 ± 817.78	< 0.001
Wash-in area under curve (iAUC)	766.04 ± 276.71	581.81 ± 394.48	0.003
Wash-out area under curve (oAUC)	1725.94 ± 763.75	946.36 ± 643.23	< 0.001
Time to peak (TTP)	24.76 ± 8.68	33.28 ± 12.17	< 0.001
Fall time (FT)	24.63 ± 6.92	24.54 ± 9.41	0.955
Wash-in rate (WiR)	1.59 ± 1.47	0.44 ± 0.37	< 0.001
Wash-out rate (WoR)	-0.58 ± 0.40	-0.22 ± 0.22	< 0.001
Mean intensity (Mean Int)	49.72 ± 24.42	31.28 ± 14.74	< 0.001
Standard deviation (STD)	33.53 ± 8.62	19.08 ± 7.95	< 0.001

Data are presented as mean ± standard deviation.

**Table 3:** Superb microvascular imaging (SMI) and contrast-enhanced ultrasonography (CEUS) variables in the region of interest (ROI) of the cervical lesions in the different groups.

	Operable (n = 19)	Non-operable (n = 45)	P value
Vascular index (VI)	21.64 ± 6.88	31.24 ± 8.93	< 0.001
Peak intensity (PI)	79.67 ± 33.14	107.33 ± 24.14	< 0.001
Rise time (RT)	15.63 ± 8.10	14.77 ± 5.31	0.615
Mean transit time (MTT)	44.39 ± 9.38	40.96 ± 10.55	0.225
Area under the curve (AUC)	2070.25 ± 919.82	2706.15 ± 914.78	0.014
Wash-in area under curve (iAUC)	615.97 ± 248.52	830.39 ± 276.41	0.004
Wash-out area under curve (oAUC)	1454.51 ± 758.30	1875.11 ± 692.19	0.035
Time to peak (TTP)	25.38 ± 6.81	25.61 ± 6.81	0.285
Fall time (FT)	25.61 ± 6.93	24.64 ± 6.01	0.574
Wash-in rate (WiR)	0.90 ± 0.51	1.93 ± 1.62	< 0.001
Wash-out rate (WoR)	-0.54 ± 0.44	-0.60 ± 0.37	0.621
Mean intensity (Mean Int)	41.42 ± 21.19	53.91 ± 24.37	0.046
Standard deviation (STD)	30.58 ± 6.95	35.31 ± 8.25	0.032

Data are presented as mean ± standard deviation.

### Quantified superb microvascular imaging analysis

Our previous study identified three distinct vascular patterns in cervical cancers based on SMI: branch-like, crab claw-like, and fireball-like (19). Among the operable group, 5 cases (26.3%) exhibited a branch-like pattern, 7 (36.8%) displayed crab claw-like patterns, and 7 (36.8%) showed fireball-like patterns. Conversely, in the non-operable group, only 2 (4.4%) cases demonstrated a branch-like pattern, while 9 (20.0%) displayed crab claw-like patterns, with the majority of cases (75.6%) presenting fireball-like patterns (P < 0.001) (**Figure 3**). These results indicated that the vascular pattern could serve as an initial differentiator between the operable and non-operable groups. Furthermore, the quantitative superb microvascular imaging (qSMI) analysis revealed that the mean vascular index (VI) of cervical cancer was (28.39 ± 9.42). Notably, the mean VI in the non-operable group was significantly higher than that in the operable group (31.24 ± 8.93 vs. 21.64 ± 6.88, P = 0.009) (**Table 3**).

### Comparison between VI and qCEUS parameters

The analysis revealed a statistically significant correlation between the parameters quantified in SMI (VI) and those obtained from TIC (qCEUS). VI exhibited a very strong or high correlation with the quantified CEUS parameters, including PI (r = 0.854, P < 0.001) and AUC (r = 0.635, P < 0.001). Moreover, a moderate correlation was observed with iAUC (r = 0.584, P < 0.001), oAUC (r = 0.595, P < 0.001), WiR (r = 0.444, P < 0.001), Mean Int (r = 0.563, P < 0.001), and STD (r = 0.543, P < 0.001). Additionally, there was a weak negative correlation with MTT (r = -0.326, P = 0.009) and TTP (r = -0.284, P = 0.023) (**Table 4**).

**Table 4:** Correlations between the VI and quantified contrast-enhanced ultrasonography parameters considering in the different groups.

Correlation of VI with	Correlation coefficient (r)	P value	Fisher’s z	95% confidence interval
Peak intensity (PI)	0.854	< 0.001	1.271	0.770 ~ 0.909
Rise time (RT)	-0.038	0.767	-0.038	-0.281 ~ 0.209
Mean transit time (MTT)	-0.326	0.009	-0.338	-0.529 ~ 0.087
Area under the curve (AUC)	0.625	< 0.001	0.733	0.448 ~ 0.755
Wash-in area under curve (iAUC)	0.584	< 0.001	0.669	0.395 ~ 0.726
Wash-out area under curve (oAUC)	0.595	< 0.001	0.685	0.409 ~ 0.734
Time to peak (TTP)	-0.284	0.023	-0.292	-0.495 ~ 0.041
Fall time (FT)	-0.044	0.731	-0.044	-0.287 ~ 0.204
Wash-in rate (WiR)	0.444	< 0.001	0.477	0.223 ~ 0.622
Wash-out rate (WoR)	-0.167	0.189	-0.169	-0.396 ~ 0.082
Mean intensity (Mean Int)	0.563	< 0.001	0.637	0.369 ~ 0.711
Standard deviation (STD)	0.543	< 0.001	0.608	0.342 ~ 0.696

**Figure 4:**
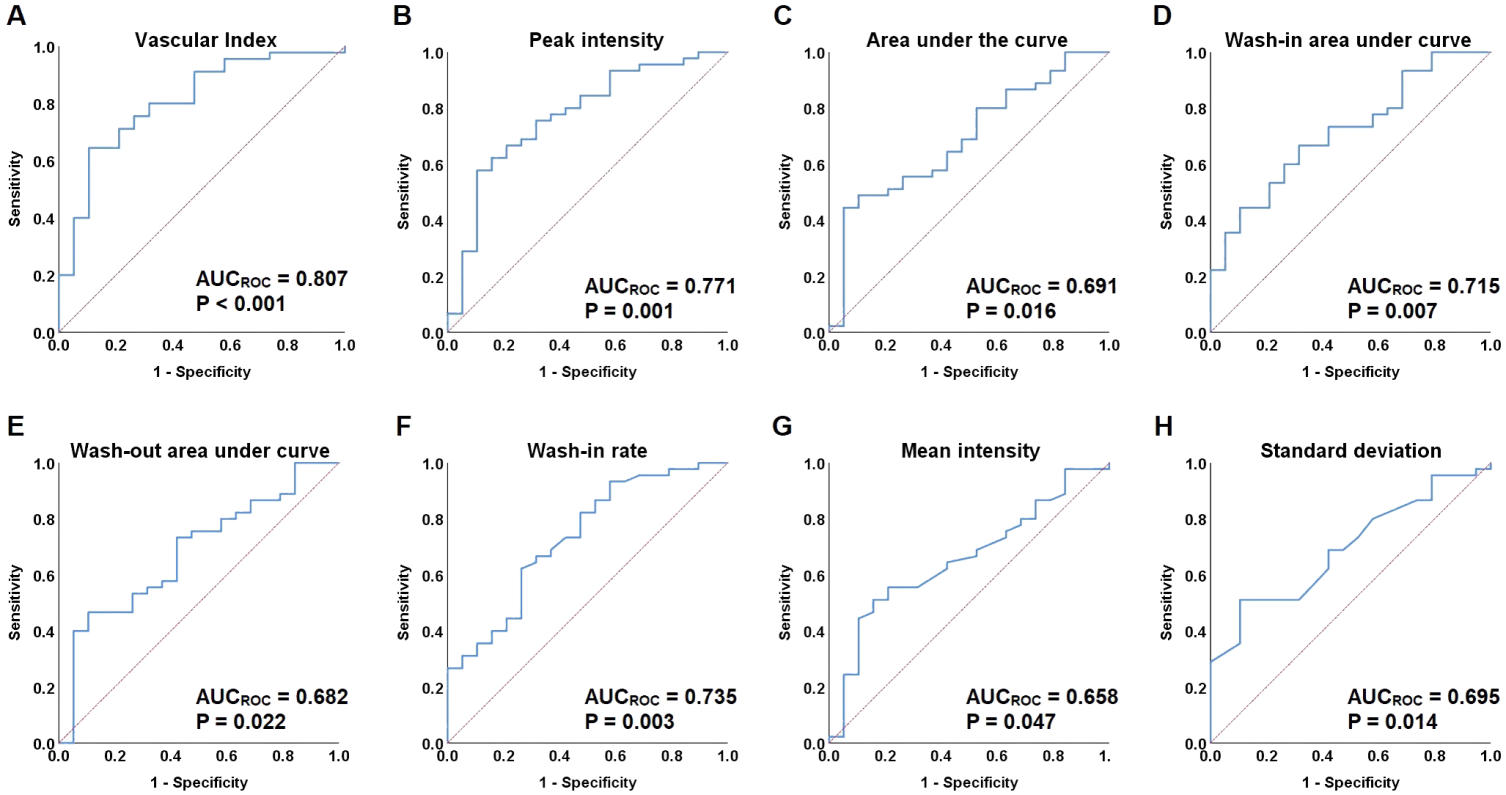
Receiver-operating characteristic (ROC) curves for vascular index (VI) and quantified contrast-enhanced ultrasonography (qCEUS) parameters. **(A)** ROC curves of vascular index (VI). **(B)** ROC curves of peak intensity (PI). **(C)** ROC curves of area under the curve (AUC). **(D)** ROC curves of wash-in area under curve (iAUC). **(E)** ROC curves of wash-out area under curve (oAUC). **(F)** ROC curves of the wash-in rate (WiR). **(G)** ROC curves of mean intensity (Mean Int). **(H)** ROC curves of standard deviation (STD).

ROC curves were constructed to assess the discriminatory ability of microvascular architecture features in cervical lesions, as measured by VI and qCEUS parameters (**Figure 4**). VI, PI, AUC, iAUC, oAUC, WiR, Mean Int, and STD demonstrated the capacity to differentiate between the operable and non-operable groups, with AUC_ROC_ values of 0.807 (P < 0.001), 0.771 (P = 0.001), 0.691 (P = 0.016), 0.715 (P = 0.007), 0.682 (P = 0.022), 0.735 (P = 0.003), 0.658 (P = 0.047), and 0.695 (P = 0.014), respectively (**Table 5**). Additionally, VI exhibited superior predictive performance for treatment-group assignment compared to qCEUS parameters, achieving a sensitivity of 64.4% and specificity of 89.5%.

**Table 5:** Receiver-operating characteristic (ROC) analysis for the predictive performance for treatment-group assignment by VI and quantified contrast-enhanced ultrasonography parameters.

Parameters	AUC_ROC_	Sensitivity (%)	Specificity (%)	Cut-off value	P value	95% confidence interval
Vascular index (VI)	0.807	64.4	89.5	28.9	< 0.001	0.691 ~ 0.923
Peak intensity (PI)	0.771	57.8	89.5	110.4	0.001	0.641 ~ 0.900
Area under the curve (AUC)	0.691	44.4	94.7	2869.7	0.016	0.552 ~ 0.830
Wash-in area under curve (iAUC)	0.715	66.7	68.4	721.2	0.007	0.583 ~ 0.846
Wash-out area under curve (oAUC)	0.682	46.7	89.5	1915.2	0.022	0.541 ~ 0.823
Wash-in rate (WiR)	0.735	62.2	73.7	1.2	0.003	0.600 ~ 0.869
Mean intensity (Mean Int)	0.658	51.1	84.2	50.5	0.047	0.516 ~ 0.800
Standard deviation (STD)	0.695	51.1	89.5	36.5	0.014	0.564 ~ 0.826

## Discussion

Tumor angiogenesis refers to the formation of new blood vessels within tumor tissue, which provides nutrients and oxygen to the tumor while also facilitating the invasion and metastasis of cancer cells (23). Studies have shown that larger tumors typically require more blood supply to meet their demands, especially in advanced cervical cancer, where there may be a greater degree of neovascularization (24, 25). Based on our understanding of the mechanisms behind cervical cancer angiogenesis, many anti-angiogenic drugs are currently in use or under development (26, 27). Therefore, clinical assessment of tumor vasculature can aid in the diagnosis of cervical cancer, selection of management strategies, and prediction of prognosis.

CEUS is a rapidly evolving technology in recent years, primarily leveraging the nonlinear acoustic effects of microbubbles to enhance the spatial resolution of grayscale imaging. It provides real-time information about tissue perfusion and metabolic status. CEUS offers advantages in detecting subtle blood vessels and low-flow blood perfusion, reflecting changes in microcirculation signals within lesion tissues. Due to the rapid growth of cervical tumors, they stimulate the formation of numerous new blood vessels, often characterized by various features such as arteriovenous shunting, lack of elasticity, weak vessel walls, high blood flow velocity, and low resistance. This leads to non-uniform vascular density in cervical tumors compared to normal tissues. There are few studies on the application of CEUS in cervical cancer. CEUS has been reported to have moderate to good agreement with MRI in assessing tumor size and local invasion in advanced cervical cancer and in assessing local staging for surgical treatment (16, 17, 28–30). All cases in this study displayed typical enhancement patterns in contrast-enhanced TIC, consistent with observations in many other highly vascularized tumors. The distinction between the operable and non-operable groups lies in the degree of cervical lesion enhancement, which can be more intuitively reflected through qCEUS parameters (including PI, AUC, iAUC, oAUC, WiR, Mean Int, STD), providing a basis for treatment selection. Other measured indicators in this study, such as RT, TTP, MTT, and FT, are related to uterine blood flow velocity. The basic hemodynamics of each uterus differ and are influenced by factors like cardiac and pulmonary resuscitation and physical condition (17). This explains why we found time-related indicators couldn’t effectively differentiate between the operable and non-operable groups. Consequently, intensity-related indicators may have the potential to distinguish early and advanced cervical cancer by evaluating tumor microvessels. However, the use of TIC for analysis is time-consuming and complex, which may pose considerable challenges for routine clinical application of qCEUS.

Ultrasonography Doppler signals originate not only from blood flow but also from tissue motion (clutter). Clutter signals overlap with low-velocity flow components. Traditional Doppler techniques employ one-dimensional wall filters to remove clutter, resulting in the loss of slow-flow components. In contrast, SMI uses multi-dimensional filters to separate flow signals from clutter, thereby eliminating clutter while preserving slow-flow signals (31, 32). While the superiority of SMI in normal perfusion patterns has not been established, it surpasses color and power Doppler ultrasonography in diagnostic efficacy by detecting extremely low-velocity blood flow within lesions. Furthermore, quantitative analysis of tumor vasculature has become possible recently through the calculation of a vascular index using dedicated software (the VI Test App from Toshiba Medical Systems Corporation). The vascular index (%) represents the ratio of Doppler signal pixels to total lesion pixels. Our previous series of studies have shown that this parameter provides valuable quantitative analysis information about the extent of vascularity, thereby improving the diagnostic rate of cervical lesions and effectively monitoring the efficacy of cervical cancer radiotherapy and chemotherapy (19, 20). In this study, SMI results demonstrated stronger signals and higher VI values in cervical lesions in the non-operable group compared to the operable group. While SMI demonstrates advantages in assessing microvasculature and detailed evaluation of vascular distribution and quantification, its limitation lies in its inability to assess tumor perfusion patterns.

Comparative studies between SMI and other methods (such as color or power Doppler ultrasonography and CEUS) conducted to date have mostly relied on semi-quantitative assessments, with results depending on the experience and skills of the researchers (33–35). To the best of our knowledge, this study is the first to compare the application value of qSMI (VI) and quantitative CEUS (TIC) in predicting treatment regimens for cervical cancer. There exists a strong statistical and significant correlation between the quantified parameters of qSMI and qCEUS. The VI and qCEUS (particularly intensity-related indicators) exhibit differences in the characteristics of blood flow within cervical cancer, distinguishing the operable and non-operable groups. ROC analysis revealed that qSMI outperforms qCEUS in terms of accuracy and sensitivity in differentiating operable and non-operable groups of cervical cancer. Recent fundamental research indicates that while SMI may not match the superiority and accuracy of vascular endothelial growth factor receptor 2 (VEGFR2)-targeted microbubble (MBVEGFR2)-based ultrasonography molecular imaging in cervical cancer microvascular imaging and angiogenesis monitoring, SMI remains a viable alternative when the use of ultrasonography contrast agents is contraindicated (36). Ultrasonography contrast remains indispensable in characterizing unclear cervical lesions, where the infiltration and outflow of contrast agents are crucial.

The study still has some limitations. Firstly, this was a single-center study with a relatively limited number of analyzed patients. Secondly, we did not compare SMI, CEUS, and microvessel density, which has been found to be associated with tumor growth and metastasis. Thirdly, CEUS and SMI were acquired using different ultrasonography devices, which could have influenced our results. Fourthly, all examinations were conducted by the same sonographer, and we did not compare differences between different operators. However, previous studies on the application of SMI and CEUS in cervical lesions have reported that inter-observer differences had little impact on the results (17, 19, 20). Lastly, standardized image acquisition and interpretation methods for both ultrasonography techniques need broader application and larger-scale studies to establish consensus on clinically practical diagnostic criteria for cervical cancer.

## Conclusion

In summary, SMI and CEUS can assist in distinguishing operable and non-operable groups by evaluating microvasculature within cervical lesions through quantitative and qualitative analyses. They enhance the diagnostic performance of conventional ultrasonography and provide valuable information for cervical cancer treatment strategies. While there is limited research on SMI currently, its diagnostic efficacy appears to be comparable or even superior to CEUS. Considering the advantages of SMI, such as not requiring contrast agent injection and reduced post-imaging analysis time, it may become an effective alternative vascular imaging technique to CEUS.

## Data Availability

The raw data supporting the conclusions of this article will be made available by the authors, without undue reservation.
